# Gustav Nikolaus Specht (1860–1940): Sein Einfluss auf die Nosologie Kraepelins und Annäherungen an seine Position im Nationalsozialismus

**DOI:** 10.1007/s00115-021-01153-6

**Published:** 2021-07-23

**Authors:** Birgit Braun, Johannes Kornhuber

**Affiliations:** 1 Abteilung für Psychosomatische Medizin, Universitätsklinikum Regensburg, Franz-Josef-Strauss-Allee 11, 93053 Regensburg, Deutschland; 2 Psychiatrische und Psychotherapeutische Klinik, Friedrich-Alexander-Universität Erlangen-Nürnberg, Schwabachanlage 6, 91054 Erlangen, Deutschland

**Keywords:** Chronische Manie, Paranoiafrage, Zeitgenössische psychopathologische Diskussion, Erbgesundheit, Exogen depressiver Reaktionstypus, Chronic mania, Paranoia, Contemporary psychopathological discussion, Hereditary health, Exogenous depressive reaction

## Abstract

**Hinführung:**

Gustav Specht steht am Anfang der Erlanger Universitätspsychiatrie. 80 Jahre nach seinem Tod untersucht der vorliegende Artikel insbesondere die Rolle Spechts bei der von Kraepelin ausgehenden psychopathologisch-nosologischen Diskussion. Trotz spärlicher Datenlage unternehmen die Autoren erstmals eine Annäherung an Spechts Positionen innerhalb der nationalsozialistischen Psychiatrie.

**Methode:**

Relevantes archivalisches Material sowie Primär- und Sekundärliteratur wurden ausgewertet.

**Ergebnisse:**

Specht wurde 1897 zum außerplanmäßigen Professor und 1903 zum ersten Ordinarius für Psychiatrie in Erlangen ernannt. Specht arbeitete die Bedeutung des manischen Elementes in der Paranoia heraus. Specht ergänzte den sog. „exogenen Reaktionstypus“ Bonhoeffers 1913 um die depressiven Zustandsbilder; er selbst war – bei fremddiagnostischem Verdacht auf zyklothymes Temperament – zweimalig exogen reaktiv depressiv erkrankt.

**Diskussion:**

Durch seine Forschungsarbeiten zum pathologischen Affekt in der chronischen Paranoia beeinflusste Specht die zeitgenössische psychopathologische Diskussion nachhaltig. Spechts Perspektivenwechsel in puncto „Erbgesundheit“ lässt sich interpretieren als Anpassung an das NS-Regime.

**Schlussfolgerung:**

Das Werk Gustav Spechts kann u. a. dazu anregen, einen interdisziplinären psychopathologischen Diskurs zu kultivieren.

## Abstract (en)

**Introduction:**

Gustav Specht (1860–1940) represents the beginning of the university psychiatry in Erlangen. 80 years after his death, this article focuses on Specht’s role in the psychopathologic nosological discussion initiated by Kraepelin. Despite the sparse data situation, the authors approach for the first time Specht’s position within psychiatry under National Socialism.

**Methods:**

The relevant primary and secondary literature as well as archival sources were evaluated.

**Results:**

In 1897 Specht was appointed supernumerary professor and in 1903 the first full professor for psychiatry in Erlangen. Specht elaborated the role of the manic element in paranoia. Specht added the depressive reaction to Bonhoeffers “exogenic reaction type” in 1913; Specht himself was in the view of some colleagues suspected of having a cyclothyme temperament and twice suffered from exogenous depressive reaction.

**Discussion:**

Specht’s research studies on the pathological affect in chronic paranoia influenced the contemporary psychopathological discussion in a sustainable manner. Specht’s change of attitude towards eugenic measures can be interpreted as an adaptation to the National Socialist regime.

**Conclusion:**

The work of Gustav Specht can stimulate the cultivation of an interdisciplinary psychopathological discourse.

## Hintergrund

Specht steht am Beginn der Abspaltung der Anstaltspsychiatrie von der universitären Psychiatrie. Im ausgehenden 19. Jahrhundert ist er konfrontiert mit der zunehmenden Tendenz der deutschsprachigen Psychiatrie hin zur reinen Gehirnpsychiatrie. „Die prominentesten Vertreter dieser Richtung waren der Wiener Ordinarius Theodor Meynert (1833–1892) sowie der Breslauer Ordinarius Carl Wernicke (1848–1905)“ [6]. Auch Specht wurde von Anton Bumm (1849–1903), einem Schüler Bernhard von Guddens (1824–1886), eingeführt in experimentell-anatomische Untersuchungen am Nervensystem. Nach 1900 war die Ära der strikten „Hirnpsychiatrie“ jedoch wieder vorbei und allmählich fand die sich parallel zur zunehmenden Bedeutung der Gehirnpathologie entwickelnde klinische Schule in Emil Kraepelin (1856–1926) ihren Höhepunkt (vgl. [25]). Specht, der unter Wilhelm Hagen (1814–1888) auch die rein psychologische Betrachtungsweise kennen und schätzen gelernt hatte (vgl. [55]), näherte sich klinisch-psychologisch der Paranoiafrage, die Kraepelin wieder in den Vordergrund der psychiatrischen Streitfrage gebracht hatte (vgl. [48]).

Der vorliegende Artikel behandelt insbesondere die Position Spechts innerhalb der von Kraepelin ausgehenden psychopathologisch-nosologischen Diskussion.

Da Specht bereits 1934, ein Jahr nach der Machtübernahme Hitlers (1889–1945), emeritiert wurde, gab es bislang kein Interesse an der wissenschaftlichen Aufarbeitung seiner Positionen innerhalb der nationalsozialistischen Psychiatrie. Trotz spärlicher Datenlage unternehmen die Autoren erstmals eine Annäherung an diesen bisher ausgesparten wichtigen werkbiographischen Aspekt.

## Spechts Leben und Werk

Gustav Nikolaus Specht wurde am 25.12.1860 als 10. Kind des Kaufmanns Adolph Gottfried Hermann Eduard Specht und seiner Ehefrau Maria Christina, geb. Will, im unterfränkischen Schweinfurt geboren. Nach dortigem Besuch der Volksschule und des humanistischen Gymnasiums nahm Gustav Specht 1879 sein Medizinstudium in München auf. Am 10.04.1880 wurde Gustav Specht „[i]m Frieden auf Grund §36 d. Ersatzordnung als dauernd militär-untauglich […] ausgemustert“ [1], so ist es festgehalten unter der Rubrik „Militärverhältnisse“ auf der Personenkarte Spechts des Universitätsarchives Erlangen. Zum Grund für Spechts militärische Untauglichkeit lässt sich nur spekulieren. Zwei Semester des 5‑jährigen Studiums verbrachte Specht in Würzburg. Er beendete sein Studium in München, wo er am 27.02.1884 seine Approbation erhielt und am 10.04.1884 zum Dr. med. promoviert wurde (vgl. [51]). Seine erste Assistentenstelle trat Specht, da „[d]er praktische Arzt […] damals noch (s)ein Ideal“ [1] war, am Bürgerspital Frankfurt a. M. an. Im Sommer 1884 ging er zu Studienzwecken nach Berlin. Auch das Wintersemester 1884/1885 verbrachte er dort, zumal er sich durch Carl Westphal (1833–1890) und Emanuel Mendel (1839–1907) zunehmend für die Fachdisziplin der Psychiatrie faszinieren ließ.

Specht kehrte nach Franken zurück, wo im mittelfränkischen Erlangen, knapp 20 km entfernt von Nürnberg, 1846 die erste bayerische Kreisirrenanstalt eröffnet worden war (vgl. [9]). Dort wurde Specht ausgebildet von Friedrich Wilhelm Hagen und Anton Bumm (vgl. [69]). „Man darf sich bei dieser Gelegenheit der gewaltigen Bedeutung erinnern, welche der Psychiatrie damals in Erlangen und früher als anderswo zukam“ [22], so der Specht-Schüler und spätere „T4“-Gutachter Berthold Kihn (1895–1964; vgl. [5, 11]). In den folgenden 18 Jahren (23.03.1885 bis 30.09.1903) arbeitete Specht in der Erlanger Psychiatrie als Assistenzarzt, in der Folge als „II. Hilfsarzt“ [1] und schließlich als Oberarzt. Im Sommer 1889 legte Specht sein Physikatsexamen als Prüfung für den ärztlichen Staatsdienst in München ab und wurde zum königlichen Oberarzt der Kreisirrenanstalt Erlangen benannt.

Die Universität Erlangen ist eine der ältesten Pflegstätten der Psychiatrie. Bereits im Wintersemester 1818/1819 hielt Johann Michael Leupoldt (1794–1874) als Privatdozent in Erlangen ein Kolleg über Geistesstörungen, wohingegen Psychiatrie erst 1862 zum Prüfungsfach in Bayern wurde. Vor diesem Hintergrund kann es – entsprechend der Archivunterlagen – als geschichtlich feststehende Tatsache gelten, dass die erste bayerische Kreisirrenanstalt „nur um der Universität willen nach Erlangen verlegt worden ist. Um diese[s] Zweck[es] willen hat man selbst die für den Kreis Mittelfranken ungünstige excentrische Lage Erlangens mit in Kauf genommen“ [1]. Nachdem bayernweit Psychiatrie neun Jahre lang Prüfungsfach gewesen war, wurde es infolge der Gleichschaltung mit Preußen 1871 wieder abgeschafft. Erst 30 Jahre später legte man mit der neuen Prüfungsordnung für Ärzte vom 28.05.1901 deutschlandweit die „Irrenheilkunde“ als Prüfungsfach fest (vgl. [41]). „Nun zeigte sich auf einmal ausgerechnet Erlangen, die alte Pflegstätte der Psychiatrie, im Rückstand“ [41], zumal „[d]ie erste selbstständige deutsche psychiatrische Klinik […] bereits 1878 in Heidelberg errichtet worden“ [41] war.

Spechts Erstlingsschrift von 1891 zur „Mystik im Irrsinn“ [52] ließ ihn, „dem akademische Bestrebungen fern lagen“ [25], so sein späterer Schüler Karl Kleist (1879–1960), in der psychiatrischen Fachwelt bekannt werden. Specht betonte, die Mystik des Philosophen, Schriftstellers und Okkultisten Carl du Prel (1839–1899; vgl. [38, 50]) decke sich nicht mit dem, was man sonst unter Mystik verstehe; Specht intendierte, „all’ den durch die du Prel’schen Auseinandersetzungen in ihren Anschauungen über psychiatrische Fragen irregeleiteten Laien ein Licht aufzustecken“ [52]. Auch im weiteren Verlauf blieb es Spechts Anliegen, dem „Aberglauben“ [61] entgegenzutreten: „Das Unkraut des Aberglaubens hat kaum irgendwo einen so günstigen Boden gefunden, wie im Traumleben, und doch ist es auch wieder derselbe duftige Stoff, aus dem die Märchendichtung und sonst die Poesie goldige Fäden gesponnen“ [61].

Die Berufung Bumms nach München im Herbst 1896 veranlasste Specht, „unverhofft in den akademischen Beruf […] einzuschwenken“ [1]. Specht zeichnete sich durch „eine längere Erfahrung in Dingen der Anstaltsleitung“ [78] aus und galt als „scharfsinniger und wissenschaftlich gut geschulter Psychiater“ [78]. Sechs Monate lang leitete Specht die Anstalt kommissarisch als stellvertretender Direktor. Zum Anstaltsdirektor wurde August Würschmidt (gest. 1919) als „praktisch bewährter Fachmann“ [25] am 01.04.1897 ernannt, Specht hingegen ab 17.03.1897 unter Beibehaltung der Oberarztstelle in der Kreisirrenanstalt nicht beamteter außerplanmäßiger „Professor für Psychiatrie und Psychiatrische Klinik“ [80]. Um eine adäquate Ausbildung der Mediziner im Fachgebiet der Psychiatrie zu gewährleisten, galt es, eine eigene Klinik mit dem entsprechenden Patientengut aufzubauen (vgl. [27]). Specht unternahm zeitintensive Verhandlungen zwischen der Universität und der Kreisvertretung von Mittelfranken.

Zur Vertiefung seiner theoretischen Kenntnisse frequentierte Specht vom 24.04. bis zum 28.05.1899 Wilhelm Wundts (1832–1920) Laboratorium für experimentelle Psychologie in Leipzig. Am 15.08.1901 heiratete Specht in Nürnberg Anna Elise Birkner, mit welcher er zwei Söhne, Hermann und Wilhelm, hatte.

**Figure 1:**
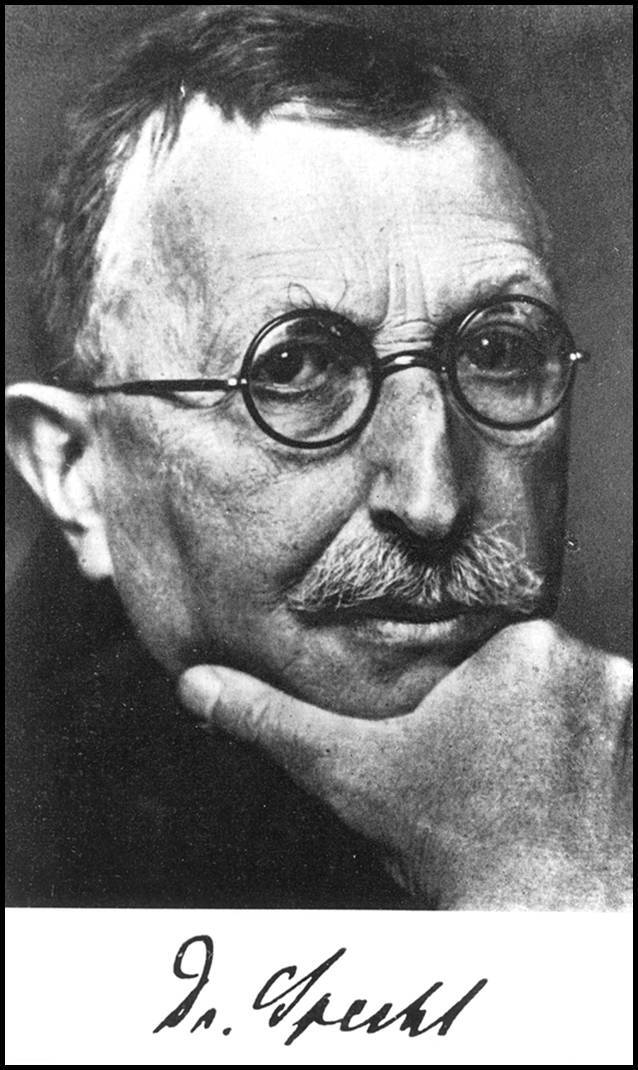

Am 29.06.1903 kam es zum Vertragsschluss zwischen der Friedrich-Alexander-Universität und der Kreisvertretung von Mittelfranken. Das Extraordinariat für Psychiatrie wurde in ein Ordinariat umgewandelt, Specht wurde am 01.10.1903 zum ordentlichen Professor ernannt – unter Aufgabe seiner Stellung als Oberarzt an der Kreisirrenanstalt. Er wurde Direktor der in einer besonderen Abteilung der Kreisirrenanstalt untergebrachten Psychiatrischen Universitäts-Klinik (Abb. 1). 1911, also in dem Jahr, in welchem der sozialpsychiatrisch aktive Gustav Kolb (1870–1938) die Nachfolge August Würschmidts als Anstaltsdirektor antrat (vgl. [10]), bezog Specht Stellung zu der vielumstrittenen Familienpflege der Geisteskranken (vgl. [10, 62]). Specht initiierte eine Vorlesung über die soziale und gerichtliche Bedeutung von „Geistesstörungen“ für Hörer aller Fakultäten. Er widmete eine Wochenstunde seiner Vorlesung der gerichtlichen Psychiatrie und etablierte ein gerichtlich-psychiatrisches Praktikum.

Spechts wissenschaftliche Auseinandersetzung mit dem Querulantenwahnsinn und sein besonderes Interesse für die gerichtliche Psychiatrie bedingten sich gegenseitig. „Das Krankheitsbild des Querulantenwahnsinns ist mehr als jede andere Form geistiger Störung ganz dazu angetan, den Richter wie den Verwaltungsbeamten bei seinen beruflichen Entscheidungen in schwere Gewissensnöte und in eine quälende Unsicherheit zu versetzen“ [63], so Specht 1912. Specht war ein gefragter Gutachter vor Gericht und referierte regelmäßig in der von ihm mitbegründeten juristisch-psychologischen Gesellschaft sowie in der mittelfränkischen Vereinigung für Psychiatrie und Neurologie, deren Initiator er war (vgl. [25]).

Nach den ersten zehn Jahren war die Erlanger Universitätspsychiatrie gut innerhalb der medizinischen Fakultät integriert und Specht stand als Prorektor an der Spitze der unter königlicher Rektoratswürde stehenden Universität. Specht ergänzte 1913 die von Karl Bonhoeffer (1868–1948) entwickelte Lehre von den symptomatischen Psychosen um die depressiven Zustände (vgl. [64]). „Wissenschaftlich war Specht in gewisser Beziehung ein Gegenspieler von Bonhoeffer“ [30], so der spätere Specht-Schüler Karl Leonhard (1904–1988; vgl. [8, 30]). Dass exogene Psychosen mit einem depressiven Syndrom einhergehen können, veranschaulichte Specht mit den Fallgeschichten einer Kohlenoxydvergiftung sowie einer Influenzamelancholie: „[b]eide Fälle haben das Besondere, dass ich dabei Beobachter und Patient in einer Person gewesen und dass ich deshalb in zweifacher Beziehung berechtigt bin, daran meine Betrachtungen anzuschließen. Es ist schon eine Reihe von Jahren her, da wurde ich durch allmählich und schubweise sich verschlimmernde psychisch-nervöse Krankheitserscheinungen in ernste Sorge versetzt […]. Nach etwa dreiwöchiger Dauer sah das Gesamtbild folgendermaßen aus: tagsüber war das Gefühl der Gedrücktheit und psychomotorischen Schlaffheit nicht mehr loszubekommen, nachts kam es zu so schweren Angstbeklemmungen, dass ich mehrmals nahe daran war, mich aus dem Fenster zu stürzen […]. Ich war eben daran, mir, weil ich mich meiner selber nicht mehr sicher fühlte, für die Nacht eine Pflegewache zu bestellen, da kam ich durch einen Zufall darauf, dass hinter dem ganzen Krankheitsbilde eine exogene Ursache stecken könne“ [64]. Specht war „Opfer einer Vergiftung mit Leuchtgas geworden“ [64], das er „während der Vorlesung aus einem versehentlich offen gebliebenen Gasrohr in refracta dosi eingeatmet hatte“ [64]. Spechts „zweite Selbstbeobachtung […] ist mit drei Worten abgemacht. Vor einigen Jahren war ich an einer schweren Influenza erkrankt und im unmittelbaren Anschluss daran stellte sich eine etwa achttägige Hypomelancholie ein, an deren Symptomgestaltung auch kein Pinselstrich fehlte“ [64]. Insbesondere verwies Specht auf das „ganz elementare, ohne jede Vermittlung trübseliger Gedanken und ohne besondere Seelenqual auftretende Gefühl des Lebensüberdrusses“ [64]. Specht betonte, er sei von körperlichem Unbehagen, Schlafstörungen und Schmerzen seelisch so wenig affiziert gewesen, dass er schon seine berufliche Tätigkeit wieder aufnahm, wobei er jedoch einen psychologisch ganz unvermittelten Zustand von Melancholie zu überwinden hatte. Von dieser Melancholie habe niemand etwas zu hören bekommen, auch seine nächste Umgebung nicht (vgl. [64]), so seine Eigenwahrnehmung. Karl Leonhard zufolge dachten manche seiner Kollegen „jedoch, dass ihm ein zyklothymes Temperament eigen sei und im Zusammenhang damit wohl ohne äußere Ursache einmal eine ernstere Schwankung depressiver Art aufgetreten sei. Daß er von zyklothymem Temperament war, kann man wohl bestätigen“ [30].

Specht, dem der Bau einer räumlich selbstständigen Klinik bereits genehmigt worden war (vgl. [22]), sah durch Kriegsausbruch seinen Bestrebungen ein vorläufiges Ende gesetzt. Er bemühte sich stattdessen um eine bessere Ausgestaltung der vorhandenen Klinik mit Fokus auf die Optimierung der gegebenen Verhältnisse (vgl. [75]).

**Figure 2:**
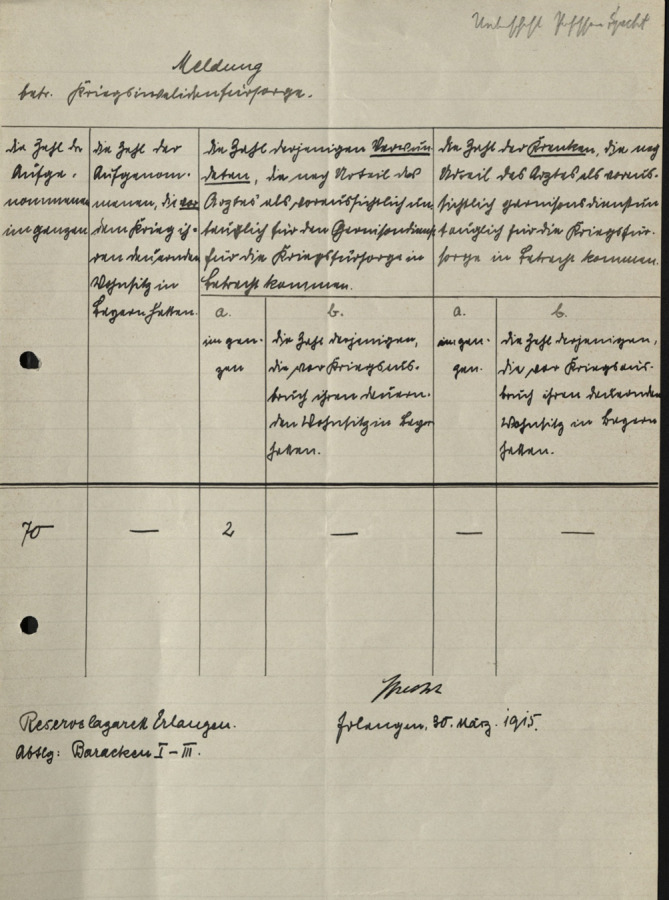

Für die Dauer seiner freiwillig übernommenen Tätigkeit im Interesse des Heeres wurde Specht am 30.12.1915 die Funktion und der Titel eines „psychiatrischen Beirates im Ehrenamt“ verliehen. In dieser Funktion oblag es ihm, zusätzlich zu seiner Tätigkeit als Ordinarius für Psychiatrie am Reserve-Lazarett Erlangen, „in besonders gelagerten Fällen im übrigen Bereich des III. Armee-Corps“ [1] Obergutachten auszuführen (Abb. 2). In seinen „historische[n] und ästhetische[n] Nebengedanken über die Erfahrungen mit den psychogenen Kriegsstörungen“ [67] von 1919 sah Specht aus den Kriegserfahrungen eine „weitere psychologische Vertiefung des scheinbar Bekannten und […] Schärfung des Blickes für bisher Verkanntes“ [67] sowie „für die Zukunft ein Ergebnis von nicht zu unterschätzendem Dauerwert“ [67] als Resultate. Kritisch positionierte sich Specht zu der in der Militärpsychiatrie angewandten „psychogene[n] Therapie“ [67]. Er sah darin eine von den üblichen Grundsätzen gravierend abweichende Behandlungsethik und einen Rückschritt hin zur „Psychiatrie vor 100 Jahren“ [67].

Mit seinem feinen Gespür für Bipolaritäten, zu dem sein verdachtsdiagnostisch unter Kollegen wahrgenommenes eigenes zyklothymes Temperament beigetragen haben mag, strebte Specht nach einer somatischen und gehirnpathologischen Fundierung des manisch-melancholischen Irreseins. Spechts psychopathologischer Schwerpunkt in der Tradition seines Lehrers Hagen erweiterte sich um eine neuro- und gehirnpathologische Betrachtungsweise in der Tradition seines Lehrers Bumm. „Dass dies nur zwei getrennte Wege zu einem gemeinsamen Ziel sind, wusste er allerdings von jeher“ [25], so Kleist. Es sei zuzugeben, „dass mit der Einbeziehung des anatomisch und physiologisch jetzt erheblich geklärten vegetativen Nervensystems in die psychiatrische Forschung wieder ein Stück Hirngebiet für die Psychiatrie gewonnen ist, und das allein ist schon der Rede wert“ [68], so Specht 1923. Im darauffolgenden Jahr schrieb er zur Beziehung von Psychovegetativum und Zwischenhirn in Ludwig Robert Müllers (1870–1962) „Lebensnerven und Lebenstriebe“ [70].

Am 06.01.1923 wurde Specht „[g]elegentlich der Wiedereinführung anerkennender Titel für verdienstvolle Gelehrte“ [15] zum Geheimen Medizinalrat ernannt. Dies war eine besondere Anerkennung seiner Forscher- und vor allem Lehrtätigkeit. Specht war mehrmals Dekan der Medizinischen Fakultät.

Nach Kleist fasste Specht nicht alles, was seine Mitarbeiter als seine „Einsichten kannten, schwarz auf weiß“ [25]. Specht hat „verhältnismäßig wenig geschrieben, aber was er schrieb, das hatte Hand und Fuß, war wohl durchdacht und in eine Form gegossen“ [15], so sein Schüler Gottfried Ewald (1888–1963). In der Tat schrieb Specht in den zehn auf seinen Beitrag zu L. R. Müllers Lehrbuch von 1924 folgenden Jahren nur 1930 einen Beitrag über das Isolieren für die Festschrift Wilhelm Weygandts (1870–1939) (vgl. [71]).

Vom 22.02.1934 findet sich der Bescheid eines Vertreters des Nationalsozialistischen Deutschen Studentenbundes, Hochschulgruppe Erlangen, an Hans Schemm (1891–1935) als Bayerischen Staatsminister für Unterricht und Kultus: „Herr Professor Specht hält trotz seines Alters noch eine sehr anschauliche und gute Vorlesung“ [2]. Am 01.04.1934 wurde Specht emeritiert, wobei er bis 30.09.1934 weiterhin die Lehrstuhlvertretung übernahm. Wie schwer sich für Specht der Rückzug aus der Klinik als seinem Lebenswerk gestaltete, beschrieb sein Nachfolger Friedrich Meggendorfer (1880–1953; vgl. [4, 7, 33]). Am 24.10.1940 verstarb Specht an einem Herzleiden.

## Specht als Wissenschaftler

Spechts bevorzugt beforschte Krankheitsentität war die Manie in allen ihren Facetten, wie der folgende Abschnitt verdeutlicht. Seine abendlichen Visiten eines unter Hagen isolierten manischen Patienten, die Specht 1930 beschrieb, mögen hierzu ein wichtiger Impuls gewesen sein (vgl. [71]).

Schon 1907 hielt er die „Angstpsychose“ für eine manische Komplikation und bezeichnete sie als „eine Mischform des manisch-depressiven Irreseins“ [57]. Als Gegenpol zur leider nur in einem Vortrag beschriebenen „stillen Manie“ [25] publizierte Specht 1908 „Über die Struktur und klinische Stellung der Melancholia agitata“ [59]: „Solch eine Kranke z. B., die lange genug davon gesprochen, wie die Angst es sei, die sie so herumtreibe und zum Sprechen anrege, konnte sich dann in einer ruhigeren Stunde selbst nicht genug darüber wundern, wie sie in all ihrer Bedrängnis eine ‚so frische Rührigkeit‘ in sich habe verspüren können“ [59]. Nach Specht verträgt sich der dogmatische Standpunkt, wonach neben der Agitation zusätzlich sichere manische Symptome wie echte Ideenflucht oder Ablenkbarkeit aufzutreten haben, nicht mit der klinischen Erfahrung. Specht beurteilte die agitierte Melancholie als Komplikation einer gemischten zirkulären Erkrankung: Im Stupor mit Angst sei eine Patientin aufgenommen worden, „nach Wochen trat Umschlag in flotte Manie ein, wenige Stunden später war die Euphorie gegen Angst umgetauscht und nun lag die reinste Melancholia agitata vor, die nach kurzer Zeit sich wieder in den Stupor mit Angst zurückverwandelte […]. Dass die soeben erwähnte Phase der agitierten Melancholie als ein Mischprodukt anzusehen ist, darüber dürfte nunmehr kein Zweifel mehr sich geltend machen“ [59]. Specht sprach dem „phantastischen Überfluss von Befürchtungen und Unglücksideen“ [59] die für eine Depression charakteristische Denkhemmung ab.

Im Jahr 1920 wurden die meisten neuaufgenommenen Patienten dem manisch-depressiven Störungsbild zugeordnet, wohingegen unter Spechts Nachfolger vor allem die schizophrenen Erkrankungen den Hauptteil der Diagnosen bildeten: „Jahrzehntelang war unter Specht das Verhältnis manisch-depressiv zu schizophrenem Formenkreis 2 zu 1. Es kehrte sich bei Meggendorfer um auf 1 zu 4“ [3]. Grund hierfür könnte sein, dass Specht die postschizophrene Depression bzw. die depressiven schizophrenen Residualzustände im manisch-depressiven Mischzustand aufgehen ließ.

Innerhalb des manischen Erkrankungsspektrums zeigte sich Specht besonders fasziniert von der chronischen Manie, so Ewald (vgl. [15]). Spechts letzte Veröffentlichung 1939, also ein Jahr vor seinem Tod, behandelt ebenfalls den manischen Krankheitszustand als das von ihm favorisierte Krankheitsbild: Sah Specht die physiologisch-chemischen sowie die endokrinologischen Arbeitshypothesen zum manisch-depressiven Irresein bislang als „nahezu ergebnislos“ [74] an, so beurteilte er die „mit hirnlokalisatorischen Erwägungen gepaarte neurovegetative Betrachtungsweise der Klinik“ [74] als aussichtsreicher. Specht schrieb „[ü]ber den vitalen Faktor im manischen Krankheitszustand“ [74] als „intim-persönliche Glückwunschgabe“ [74] an seinen „lieben Freund und ehemaligen Erlanger Arbeitskameraden Karl Kleist“ [74] zum 60. Geburtstag. Vitalität als Lebensenergie ist die vom Vitalismus angenommene besondere, dem Lebendigen innewohnende Kraft, „welche die Erscheinungen des Lebens bewirkt, das Leben erhält und in seiner geistigen Dimension gestaltet“ [37], wobei sie in ihrer Größe und Ausprägung deutlichen individuellen Unterschieden unterliegt (vgl. [37]). Specht schilderte, wie er „den Begriff der Vitalität […] zurechtgelegt und […] auf die klinische Praxis anzuwenden [s]ich gewöhnt habe“ [74]. Anhand einer Kasuistik verdeutlichte Specht, dass die „Erholungsfunktion“ [74] bei manischen Patienten nicht in den Regenerationsprozessen des Schlafes stattfinde. Um mit einem derartigen klinischen Faktum naturwissenschaftlich umzugehen, führte Specht den „in der Persönlichkeitsbewertung wieder mehr und mehr eingebürgerten Begriff der Vitalität“ [74] an. Specht sah in der gesteigerten Vitalität einen integrierenden Bestandteil des manischen Symptomenkomplexes. Im Laufe der Jahre habe er gelernt, die trotz langjähriger Alkoholkrankheit körperlich und geistig wenig affizierten Patienten als chronische Maniaci zu diagnostizieren.

Spechts wissenschaftlicher Fokus auf den pathologischen Affekt macht auch seinen Zugang zur Paranoiafrage aus. Specht sah die chronische Manie in der psychiatrischen Praxis zumeist lediglich missbraucht als „verschwommene Verlegenheitsdiagnose“ [53]. Nach Specht bildet der hypomanische Symptomenkomplex „den Kern der psychopathischen Erscheinungen“ [53], die im Rahmen der chronischen Manie auftreten. Das sekundäre Auftreten von „chronisch gereizte[r] und expansive[r] Stimmungsrichtung“ [53] auf der einen Seite und „Renommistereien“ [53] auf der anderen Seite können nach Specht „allmählich das täuschende Aussehen paranoischer Wahnbildungen annehmen“ [53]. Specht forderte eine „subtile differentiell-diagnostische Unterscheidung insbesondere dieses sekundär veränderten Krankheitsbildes von anderen chronischen, vor allem paranoischen Störungen, als ein wissenschaftliches und praktisches Postulat“ [53]. Der habituelle Grundaffekt bei der chronischen Manie – „sei er bis dahin ausgesprochen euphorisch oder überspannt oder sonst wie rein exaltativ gewesen“ [60] – nehme „in periodischer Wiederkehr und völlig endogen einen morosen, zornmütigen oder misstrauischen Charakter an“ [60]. Specht betonte, „in allen Phasen des zirkulären Irreseins“ [60] träten „wenigstens Ansätze zu paranoischer Gedankenrichtung“ [60] auf.

Zwar räumte Ewald ein, Specht mag „hier manchmal zuviel getan und zuviel gesehen haben“ [15], er schrieb Specht jedoch das Verdienst zu, die Bedeutung der chronischen Manie, insbesondere innerhalb des Querulantentums, so klar erkannt und so plastisch herausgestellt zu haben, dass „selbst ein Kraepelin“ [15] ihm gegenüber nachgeben und den Querulantenwahn umwandeln musste in den Typus des chronisch-manischen Querulanten. Auch wenn Spechts Versuch der Paranoiaintegration in die Krankheitsentität des manisch-depressiven Irresein scheiterte, so hat er gemäß Ewald „doch der Bedeutung des manischen Elementes in der Paranoia als Erster Geltung verschafft […], ein Jahrzehnt seiner Zeit vorauseilend“ [15].

Das Wesentliche in der Paranoia nach Emil Kraepelin war die Erhaltung der Persönlichkeit und das Paradigma des Querulantenwahns (vgl. [47]). Genau zu dem Zeitpunkt, als sich die Untersuchungen und Arbeiten Kraepelins mehr und mehr durchsetzten und zu Umwälzungen auf psychiatrischem Gebiet führten, wandte sich Specht der Untersuchung der Paranoiafrage und der damit eng assoziierten Wahnbildung zu. Mit Recht schrieb Josef Klüber (1873–1936) als Direktor der Heil- und Pflegeanstalt Klingenmünster und einstiger Specht-Schüler (vgl. [73]) in der Festschrift für Gustav Specht „abgeschlossen am 25.12.1930“ [26], also direkt am Geburtstag des Jubilars, Folgendes: „Als mich – es war im letzten Jahrzehnt des vorigen Jahrhunderts – mein verehrter Lehrer Gustav Specht – in die Geheimnisse der theoretischen und praktischen Psychiatrie einführte, da bot das Gebäude unserer Wissenschaft noch ein wesentlich anderes Bild wie heute. Ich darf nur daran erinnern, dass damals E. Kraepelin erst einige Zeit in Heidelberg lehrte und die ersten Auflagen seines Lehrbuches schrieb, dass also seine Lehren noch nicht Allgemeingut der deutschen Psychiatrie geworden waren. Welche Wandlung haben wir seitdem in unserer Wissenschaft erlebt!“ [26] Den Kampf um den Paranoiabegriff bezeichnete Klüber auch 1930 als noch nicht abgeschlossen (vgl. [14, 26, 28, 39]), Oswald Bumke (1877–1950) z. B. nenne die Frage der paranoiden Erkrankungen „eine der schwierigsten der klinischen Psychiatrie“ [13].

## Zeitgenössische psychopathologische Diskussion

Specht widmete sich intensiv dem Krankheitsbild der Manie und dem chronischen Querulantentum, womit er bald nach der Jahrhundertwende in relativ rascher Abfolge bis 1912 mitten im Feld der Paranoiadiskussion agierte.

Der ursprüngliche Paranoiabegriff entwickelte sich aus der einstigen „sekundären Verrücktheit“ [20], wobei das Wesentliche der Prozesscharakter war (vgl. [43]). Der Krankheitsbegriff der „Verrücktheit, der Paranoia“ [20] hatte „in den Jahrzehnten seit seiner Einführung in die Psychiatrie eine Verschiedenartigkeit seiner Um- und Abgrenzung erfahren, wie kaum ein anderer“ [20], so Werner Gutsch 1918 als „Assistenzarzt der Reserve“ [20]. Wilhelm Griesinger (1817–1868) betonte bei der Paranoiafrage einen psychischen Schwächezustand als ätiologischen Faktor, Carl Westphal (1833–1890) hingegen sah gemäß der Mendel-Ziehen-Definition der Paranoia hauptsächlich Wahnideen und Sinnestäuschungen als klinische Symptome relevant und stellte die Hypothese zum primären Ursprung der Paranoia auf (vgl. [79]). Der Stand der Paranoialehre vor Specht beinhaltete zwei Hauptrichtungen.

Die eine wurde repräsentiert vom Psychiater und Neurophysiologen Eduard Hitzig (1838–1907), der die Lehre Westphals von der Paranoia als primärer Verstandeskrankheit übernommen hatte. Der mit seinem Lehrer Westphal zerstrittene (vgl. [6, 76]) Wernicke lehrte, der Querulantenwahnsinn knüpfe ätiopathogenetisch immer an ein besonders affektvolles Erlebnis oder an eine Reihe derartiger Erlebnisse mit überwertigen Ideen an. Carl Moeli (1849–1919), erster Direktor der 1893 eröffneten zweiten Berliner Irrenanstalt Herzberge und einstiger Schüler Westphals, konnte seine Zweifel an der Grundlehre Westphals bei der Berliner Paranoiadiskussion von 1894 unter Experten nicht geltend machen, sodass auch bei dieser Gelegenheit noch die Lehre Westphals von der Paranoia als einer Verstandesstörung im Sinne eines Dogmas anerkannt wurde.

Die zweite, ältere Hauptrichtung fokussierte auf die genetische Affektanomalie, wobei der Anstaltsdirektor von Illenau, Heinrich Schüle (1840–1916), ein wichtiger Repräsentant war und die Genese des Querulantenwahns insbesondere aus moralischer Minderwertigkeit und aus angeborenem Misstrauen mit starker Affekterregbarkeit ableitete. Diesen manischen Zug unterstrich ebenso Max Koeppen (1859–1916), ein Schüler von Friedrich Jolly (1844–1904). Auch der gebürtige Nürnberger und Wernicke-Schüler Karl Heilbronner (1869–1914) sprach, so Klüber, von einer charakterogenen „Wahnbildung des Querulanten, die den hysterischen Einfällen und der pathologischen Lüge nahestehe“ [26], sich hingegen klar unterscheide von dem chronischen Krankheitsbild einer Verrücktheit im Sinne der damaligen Paranoialehre.

Hier setzte auch Specht mit seiner engen Verbindung von Querulantenwahn und manisch-melancholischem Irresein bzw. chronischer Manie an. In Weiterführung von Schüle zeigte Specht, dass beim manisch Erkrankten bereits durch seine Reizbarkeit, seinen Tatendrang, seine Vielgeschäftigkeit und sein gesteigertes Rechtsgefühl die querulatorische Tendenz angelegt sei (vgl. [26]).

In Abgrenzung zu Hitzig sah der Kraepelin-Schüler Robert Gaupp (1870–1953) 1903 enge Beziehungen der Wahnerkrankung zu einer präpsychotischen Persönlichkeit (vgl. [19, 20]), womit sich Übergänge zur Lehre Spechts abzeichnen, wonach das manisch-melancholische Irresein, vor allem die manische Komponente, zum „Querulantenwahn“ führen könne.

„Als G. Specht mit seiner Abhandlung über die affektive Genese der Wahnbildungen im Jahre 1901 an die Öffentlichkeit trat, fand er zunächst wenig Widerhall“ [15], was Ewald darauf zurückführte, dass Specht seiner Zeit voraus gewesen sei (vgl. [15, 53]).

Während Kraepelin den sog. Querulantenwahn als Vorbild der echten Paranoia sah, stellte Specht 1901 klar heraus, wie der Wahn eines chronisch manischen Patienten dauernd „am Gängelband der pathologischen Stimmung hängen“ [53] bleibt. Specht hinterfragte die bisherige Auffassung der Paranoia als eine primäre Verstandeskrankheit. Er sah vielmehr in ihr ein „primär gestörtes Affektleben“ [53]. Als limitierenden Faktor seiner Betrachtungen gestand Specht die fehlende Empirie bezüglich der Affektgenese des paranoischen Wahnes ein, zumal die Stimmungsauffälligkeit neben der Wahnidee eine separate Erscheinung sein könne. 1905 betonte Specht, das Symptomenbild der chronischen Manie sei bisher hauptsächlich in der chronischen Paranoia aufgegangen (vgl. [54]). Auf der bayerischen Psychiaterversammlung in München 1907 trat Specht in der Diskussion den Ausführungen von Karl Willmanns (1873–1945), der den Querulantenwahn als eigenartige Entwicklungen einer psychopathischen Persönlichkeit ansah, entgegen: „Es gibt eine gar nicht kleine Gruppe von Paranoikern, die unmöglich in der Dementia-praecox-Gruppe Platz finden können. Die echten und rechten Paranoiabilder gehören – den klinischen Nachweis werde ich nachher erbringen – in die Gruppe des manisch-melancholischen Irreseins. Zu dieser Anschauung bin ich natürlich nicht gekommen, weil das manisch-melancholische Irresein so in der Moderichtung liegt, sondern Schritt für Schritt hat mich seit Jahren die klinische Erfahrung dazu gedrängt“ [58]. 1908 fasste Specht auf der Jahresversammlung des Vereins bayerischer Psychiater zu Erlangen, als dessen Ehrenmitglied seine Personenkarte im Universitätsarchiv ihn auszeichnet (vgl. [1]), seine Publikationen und Anschauungen über seine Paranoiaauffassung zusammen: Die manischen Symptome der Paranoiker seien nicht bloße Begleiterscheinungen, vielmehr sei die Manie das Substrat für die Entstehung des paranoischen Zustandsbildes. Den Querulantenwahn solle man daher klinisch nicht „Querulanten-Paranoia“ [60], sondern passender „Querulanten-Manie“ [60] bezeichnen. Als Hagen-Schüler (vgl. [21]) betonte Specht, die Wahnidee stütze sich auf eine krankhaft gefälschte Erfahrung, bei welcher der Kranke subjektiv ubiquitär zu viel hinter den Erscheinungen der Außenwelt im Sinne einer „krankhafte Eigenbeziehung“ [60] suche; das Misstrauen als „Mischgefühl“ [60] prädisponiere hierfür. Spechts „biologisch orientierte Hypothese eines für das Krankheitsbild der Paranoia konstitutiven manischen Elementes“ [44] führte ihn zur Auffassung der Paranoia als affektpsychotischen Mischzustand. So hielt Specht 1908: „nach wie vor daran fest, dass der pathologische Affekt auch für den spezifischen Paranoiawahn eine conditio sine qua non darstellt“ [60].

Kraepelin stimmte Specht damals teilweise zu, z. B. dass Wahnbildung Primordialsymptom des Krankheitsbildes sei. Nach Kraepelin jedoch führte eine krankhafte Verstandestätigkeit – nicht ein pathologischer Affekt – zur Wahnbildung. Für den paranoischen Wahn relevant beurteilte Kraepelin damals insbesondere eine psychopathische Veranlagung. In seinem Vortrag „Über paranoide Erkrankungen“ [29] auf der Versammlung Bayerischer Irrenärzte in Regensburg betonte Kraepelin am 29.06.1912 schließlich, die Selbstständigkeit der Gruppe krankhafter Persönlichkeiten könne im Sinne Spechts angefochten werden, da sie der chronischen, besser wohl der konstitutionellen Manie mit ihren paranoiaähnlichen Zuständen zuzurechnen sei (vgl. [29]). Brodschöll und Strotzka sahen 1957 das „Faszinierende am Paranoiaproblem“ [12] darin, dass hiermit erstmals über die Psychogenie der sog. „endogenen Psychosen“ [12] diskutiert worden sei. Spechts Beschreibung des chronisch manischen Querulanten, bei welchem „[d]ie Entstehung des psychopathischen Zustandes […] wohl immer mit der Ausreifung der Persönlichkeit zusammenfallen [wird]“ [53], mag wesentlich dazu beigetragen haben, dass Kraepelin „schließlich auch den Querulantenwahn von der Paranoia abtrennte und in die Gruppe der psychogenen Erkrankungen einreihte“ [20].

Gewissermaßen als Antipode zu Kraepelin trat Specht auch mit seinen wichtigen Anregungen und klärenden Gesichtspunkten von 1913 im Fragenkomplex um den sog. „exogenen Reaktionstypus“ Bonhoeffers auf (vgl. [64]), die er um die depressiven Zustandsbilder ergänzte, wie im biographischen Abschnitt aufgezeigt. Kraepelin hatte irrtümlich gelehrt, dass jede psychoseinduzierende exogene Schädigung von Gehirn oder Organismus eine ganz bestimmte Geistesstörung bewirke, sodass eine ausgereifte Differenzialdiagnostik das Unterscheiden einer alkoholinduzierten von einer kokaingetriggerten Psychose sowie einer Typhuspsychose von einer Paratyphus-A-Psychose ermöglichen müsse.

Seine Rede beim Antritt des Prorektorates 1913 gestaltete Specht antizipatorisch mit dem ernsten Thema „Krieg und Geistesstörung“ [65].

Der wissenschaftliche Diskurs um die traumatische Neurose bis zum Ersten Weltkrieg wurde außerordentlich intensiv geführt (vgl. [34]). Einerseits zielte er ab auf akute oder chronische seelische Erschütterung im Anschluss an unmittelbar lebensbedrohliche Katastrophen im Sinne der Schreck- oder Panikreaktion. Andererseits bezog er sich auf einen Symptomenkomplex, bei dem sich in der Folge eines Traumas ohne organische Schädigung – allein unter dem Eindruck einer „Begehrensvorstellung“ – eine Neurose entwickelt. Während seiner Leitung des Berliner Militärkrankenhauses für Nervenkrankheiten behandelte Hermann Oppenheim (1858–1919) „Kriegszitterer“ (vgl. [35]). Er führte deren Symptome auf traumabedingte seelische Erschütterungen zurück, die zu funktionellen Störungen des Gehirns führen, welche möglicherweise wiederum durch Umlagerungen auf molekularer Ebene bedingt seien (vgl. [34, 35]). Die Auffassung Oppenheims wurde jedoch von den meisten anderen Psychiatern nicht gestützt. Sie sahen vielmehr eine „Willensschwäche“, welcher man mit rigoroser Disziplin begegnen solle, um die Soldaten möglichst bald wieder fronttauglich zu machen. Bei der 8. Jahresversammlung der Gesellschaft Deutscher Nervenärzte in München 1916 standen sich die Konzepte „traumatische Neurose“ vs. „Willensschwäche“ eklatant gegenüber (vgl. [16]).

Betrachtet man das aktuell intensiv beforschte Phänomen der posttraumatischen Belastungsstörung bei Soldaten im Kriegseinsatz (vgl. [82]), so ist zu betonen, dass dies am ehesten dem von Specht unter dem Begriff „Emotionspsychosen“ [65] subsumierten Krankheitsbild entsprechen könnte, wobei eine tendenziöse Diagnostik immer auch limitierend eine ahistorisch-retrospektive Perspektive einnimmt. Als „Emotionspsychosen“ [65] bezeichnete Specht posttraumatische Reaktionen auf Kriegserlebnisse mit „gewöhnlich stürmisch einsetzende[n] hysterische[n], epileptische[n], delirante[n] oder halluzinoseartige[n] Paroxysmen“ [65]. „Man hat schon gesagt, wer nicht psychopathisch disponiert ist, wird auch im Krieg nicht geisteskrank. Das ist […] einerseits eine Trivialität, andernteils nicht wahr, denn unter Strapazen […] kann auch der ‚Gesündeste‘ aus dem Geleise geworfen werden“ [65]. Specht zeigt sich also in seiner Position zur rein kriegsereigniskorrelierten psychischen Störung bereits 1913 sehr fortschrittlich – ganz im Gegensatz z. B. zur Ansicht, die Friedrich Panse (1899–1973) als Begründer der „Pansen“-Methode zur Wiederherstellung der Fronttauglichkeit noch 1940 vertrat (vgl. [36]).

## Specht in der NS-Zeit

Gemäß Friedrich Meggendorfer hatten Spechts Konzepte „die erbbiologische Forschung befruchtet“ [33]. So fundierte Specht z. B. seine Auffassung der Paranoia als Mischbild des manisch-melancholischen Irreseins durch Hinzuziehen von Erblichkeitsfragestellungen unter Nachweis manisch-melancholischer Erkrankungen in „Sippen“ [33] von Paranoikern und Querulanten. Specht sah 1908 „die Melancholien und Manien nicht minder wie die Paranoia der Hauptsache nach auf dem Boden der Veranlagung entstanden“ [60]. Nach Meggendorfer lehrte Specht Wichtiges in Bezug auf die prognostische Einschätzung von Psychopathien: „Das Zyklothyme, insbesondere das Manische, wirkt günstig, konservierend, es berechtigt zu einer guten Prognose; das Schizotyhme ist ungünstig, es wirkt destruierend, gestaltet die Prognose ungünstig“ [33]. Specht führte die spezifische Heredität als Beweismaterial für seine Paranoialehre an: Er habe in den Familien einer Reihe von Paranoikern „alle möglichen Varianten des manisch-melancholischen Irreseins feststellen können“ [60]: „eine mit einem gesunden Mann verheiratete Frau, bei der Moral insanity (vgl. [32]) diagnostiziert war, die aber tatsächlich an chronischer Hypomanie mit periodischen Schwankungen gelitten hatte, hatte drei hochbegabte Kinder von denen die zwei Töchter paranoisch wurden und der Sohn ausgesprochen manisch-melancholisch, er endete frühzeitig durch Selbstmord. Ein Mann, der an sogenannter Querulantenparanoia litt, stammte von einem chronischen Hypomaniker ab und hatte einen Sohn, der deutlich manisch-depressiv war, usw. Ähnlich sind meine sonstigen einschlägigen Erfahrungen“ [60].

Specht förderte die Erbbiologie, indem er die Untersuchungen seines Mitarbeiters W. Medows „Zur Erblichkeitsfrage in der Psychiatrie“ [31] anregte. W. Medow untersuchte in seiner Arbeit als Assistenzarzt an der Psychiatrischen Klinik Erlangen 1914 die Frage, „ob durch systematische Nachkommenschaftsverhinderung geistig abnormer oder geistig gestörter Personen die Möglichkeit eines Erlöschens oder eines wenigstens als wesentlich zu betrachtenden Rückganges geistiger Erkrankungen oder Abnormitäten zu erwarten“ [31] ist. Als Probanden hatte er „sämtliche während eines Jahres auf der Männerstation der Klinik befindliche Kranke bezüglich ihrer Heredität untersucht, ohne eine Auswahl zu treffen“ [31]. Nach Medows Befunden „scheint mindestens in der Hälfte der Fälle eine direkte gleichartige Vererbung des reaktiv psychopathischen Grundzustandes stattzufinden, worin sich ebenfalls eine Analogie zu den manisch-melancholischen Störungen“ [31] finden lasse. Während bislang nach Medow bei manisch-depressivem Irrsein die Beweismittel der erblichen Verhältnisse wenig herangezogen wurden, habe Specht als einziger „unter Verweis auf die psychopathische Durchseuchung der Geschlechter“ [31] bereits die erbliche Belastung hervorgehoben. Medow schloss sich ganz den Ansichten Robert Sommers (1864–1937) und Bumkes an, „welche die Sterilisation als Mittel im Kampfe gegen die Geisteskrankheiten ebenfalls zurückweisen“ [31] – so zumindest der Stand 1914.

Beim kritischeren Teil der psychiatrischen Fachwelt werde „der vielgehörte Ruf nach gesetzlicher Regelung des Schutzes der Nachkommenschaft vor erblich-degenerativer Belastung kein williges Ohr“ [66] finden, so Specht 1916. Mit Verweis auf Nordamerika, „wo in vielen Staaten bereits derartige Gesetze bestehen, in denen sogar die Sterilisation psychisch degenerativer Individuen vorgesehen ist, so lockt gerade dieses Beispiel nicht zur Nachahmung“ [66]. Auf eine Pro- und Kontraargumentation verzichtete Specht 1916 unter Verweis darauf, dass eine praktische Inanspruchnahme vergleichbarer Maßnahmen hierzulande „bis auf weiteres nicht zu denken ist“ [66]. Der Rassenhygiene dienende operative Eingriffe seien aber auch aus individuell-therapeutischen Gründen vorgeschlagen und bereits mehrfach – und zwar nicht nur in Nordamerika – ausgeführt worden: „Mit der Sterilisation hat man z. B. schwachsinnige Kindsmörderinnen vor der Wiederholung ihrer Verbrechen, moralisch haltlose Imbezile vor Schwangerschaften geschützt und ihnen damit eine dauernde Anstaltsinternierung erspart. Mit der Kastration will man nicht nur die Dämpfung eines krankhaften Geschlechtstriebes, sondern auch eine Besserung des psychischen Gesamtzustandes bewirkt haben. Für unsere Verhältnisse kommen solche Eingriffe bis auf weiteres praktisch nicht in Betracht, da die rechtlichen Voraussetzungen dazu noch fehlen“ [66].

Implizit kommt an dieser Stelle zum Ausdruck, dass Specht bei Vorhandensein der entsprechenden rechtlichen Voraussetzungen durchaus die Praxis der eugenischen Sterilisation und der forensisch-eugenischen Kastration in näheren Betracht zog.

Medow zeigt sich als Anhänger positiver Eugenik im Sinne von „Vorsicht in der Eheschließung mit allzu gefährdeten Individuen und Förderung der naturgemäßen Hygiene und Kampf gegen die keimschädigenden Gifte“ [31]. Folgende Aussage Spechts könnte ihn primär sogar als Skeptiker bezüglich positiver Eugenik erscheinen lassen: „Übrigens wäre zu bedenken, ob der gewissenhafte Arzt statt die immerhin nur möglichen Folgen psychiatrisch nicht ganz einwandfreier Eheschließungen allzu ernst zu nehmen, seinen prophylaktischen Beruf nicht besser erfüllt, wen er sich zum zukunftsfrohen Prediger des Regenerationsgedankens macht“ [66]; es gäbe ohnehin genug Familien, in denen der chronische Druck der Erblichkeitsfrucht für die Seelen eine schlimmere Belastung bedeute wie die ererbte Keimesanlage (vgl. [66]). Bei den Schizophrenien beurteilte Specht den Erbgang noch als „zu unübersichtlich, um für ein wirksames Eheregulativ verwertet werden zu können“ [66].

Eine weitere Analyse Spechts „erbgesundheitlicher“ Untersuchungen und Aussagen in seinem Lehrbuch von 1916 lassen ihn weniger skeptisch, sondern vielmehr eugenikaffin erscheinen. So riet er von allzu frühem Heiraten ab, „weil eine große Anzahl von konstitutionellen Psychosen erst in der zweiten Hälfte der Zwanziger zum Ausbruch zu kommen pflegt“ [66]. Specht betonte an dieser Stelle, es gebe bereits gewissen gesetzlichen Rückhalt in Form der vorsorglichen Entmündigung, der Ehescheidungsnichtigkeits- und Anfechtungsklagen, welche „einen gewissen Schutz gegen psychiatrisch bedenkliche Kindererzeugung“ [66] „immerhin von Fall zu Fall“ [66] gewähren würden [66]. Kasuistiken hierzu führte Specht 1935 weiter aus (vgl. [72]). Innerhalb des psychopathischen Erkrankungskreises ließ Specht den klinisch wesensgleichen Formen ein schwereres Belastungsgewicht zukommen: „Anders ausgedrückt, neigt man jetzt mehr zur Annahme, dass nicht eine spezifische Veranlagung zu geistiger Erkrankung überhaupt, sondern eine spezifische Veranlagung zu besonderen klinischen Krankheitsformen vererbt wird“ [66]. Wenn Kleist dafür plädierte, die „Psychopathie“ solle unter das Gesetz zur Verhütung erbkranken Nachwuches (GzVeN) fallen (vgl. [42]), um einer Vermehrung der „weit gefährlicheren intelligenten psychopathischen Gemeinschaftsschädlinge“ [46] entgegenzuwirken, so könnte dies auch die – der geänderten Gesetzgebung angepasste – Meinung seines ehemaligen Lehrers Gustav Specht wiedergeben.

Im Rahmen der zunehmend an Bedeutung gewinnenden rassenhygienischen Bestrebungen änderte sich nämlich Spechts Position in Bezug auf erbhygienische Maßnahmen. Hatte Specht 1914 die distanzierte Betrachtungsweise Medows in Bezug auf eugenische Maßnahmen gefördert, so ließ er später seinen Oberarzt Berthold Kihn ungehindert regimeangepasst eine negative Eugenik und sogar „Euthanasie“ propagieren. In seinem Empfehlungsschreiben für Kihn betonte Specht, dieser habe der Erbgesundheitslehre „1931 schon in einer Studie über die Ausschaltung der Minderwertigen und in Bearbeitung der choreatischen Erscheinungen Beachtung gezollt“ [1]. An dieser Stelle datierte Specht die Publikation Kihns zeitlich um ein Jahr zurück (vgl. [23, 24]). Ob dies bewusst strategisch war, muss offen bleiben. Hatte Specht 1916 den Erbgang bei den Schizophrenien noch als zu unklar angesehen, um positive Eugenik anwenden zu können, so vergab er sogar noch als Emeritus ein Dissertationsthema über „[d]ie Nachkommen von Schizophrenen und das GZVeN“ [17]: „Es sind die phänotypisch Gesunden aber Keimträger, aus denen die Schizophrenie sich immer erneuert“ [17]. Diese seien nicht dem GzVeN unterworfen, sodass weitere Hilfsmittel „im Vorbeugekampf gegen die Schizophrenie“ [17] notwendig seien. Den Anfang hierzu sah Spechts Doktorand Fuchs in den gemäß GzVeN-Ergänzungsgesetz vom 26.06.1935 gesetzlich geregelten Schwangerschaftsunterbrechungen sowie der verkürzten Einspruchsnotfrist. Hierdurch könnten auch „vereinzelte Fälle von Schizophreniekandidaten“ [17] erfasst werden.

Eventuell könnte Specht durch sein als Emeritus betreutes Promotionsthema politische Gunst erworben haben: Hatte sich das Bayerische Staatsministerium für Unterricht und Kultus am 18.12.1935 gegen eine Gratulation zu Gustav Spechts 75. Geburtstag entschieden, so findet sich eine Notiz vom 02.10.1939, wonach die Übersendung eines „Glückwunschschreibens (zum 80. Geburtstag)“ [1] veranlasst worden sei. „Bedenken wegen der früheren und jetzigen politischen Haltung sowie wegen der Abstammung und der früheren Logenzugehörigkeit bestehen nicht“ [1]. Ob Gustav Specht tatsächlich zu seinem 79. Geburtstag von Seiten des Bayerischen Staatsministeriums für Unterricht und Kultus verfrühte Glückwünsche zum 80. Geburtstag erhielt, lässt sich nicht rekonstruieren.

Spechts Perspektivwechsel in puncto „Erbgesundheit“ lässt sich interpretieren als Anpassung an das NS-Regime mit seiner Legalisierung eugenischer Interventionen.

Interessant zeigt sich auch Spechts zunehmend versöhnliches Verhalten gegenüber Johannes Reinmöller (1877–1955), dem Direktor der Erlanger Universitäts-Zahnklinik und Rektor der Friedrich-Alexander-Universität von 1933 bis 1935. 1931 war es zum Eklat zwischen Specht und Reinmöller bei den Senatssitzungen gekommen (vgl. [1]): Am 04.04.1931 ließ sich Specht beim Rektor der Erlanger Universität aufgrund von Unstimmigkeiten mit Reinmöller „für alle Senatssitzungen als entschuldigt betrachten“ [1]. Am 01.07.1931 begründete Specht sein Vorgehen detaillierter: „Auch davon will ich nichts Näheres verraten, welch’ unerhörte Szenen wir schon seitens Reinmöllers in der Fakultät über uns ergehen lassen mussten. Man kann schlankweg behaupten, dass Ähnliches in keiner Fakultät Deutschlands geduldet worden wäre. Zu seiner Entschuldigung habe ich schon vor Jahren einmal in der Fakultät eine psychiatrische Begutachtung seines Geisteszustandes abgegeben. […] So übt Prof. Reinmöller wie in der Fakultät so im Senat einen terrorisierenden Einfluss aus, der jede sachliche Aussprache unmöglich macht“ [1]. Die Aussage Spechts, er habe Reinmöller psychiatrisch begutachtet, lässt sich nicht auf Richtigkeit prüfen. Ein entsprechend schriftlich fixiertes Gutachten konnte in den eingesehenen Archivbeständen nicht gefunden werden. 1937 sandte Specht seinem einstigen Widersacher Reinmöller ein Geschenk zum 60. Geburtstag (vgl. [1]). Diese Geste könnte hinweisen auf Spechts Bemühen um eine späte Aussöhnung. Alternativ könnte sie Spechts Buhlen um parteiliche Protektion durch das NSDAP-, SA- und SS-Mitglied Reinmöller symbolisieren. War Specht im Kaiserreich Mitglied der national liberalen Partei, so gehörte er zur Zeit der Weimarer Republik der linksliberalen Deutschen Demokratischen Partei an. Die Personenkarten-Felder „Mitglied der NSDAP seit“ [1] und „MitgliedsNr“ [1] zeigen sich bei Specht leer.

## Diskussion

Während Gustav Specht heutzutage – ganz zu Unrecht – unter deutschen Psychiatern und sogar Psychiatriehistorikern wenig bekannt ist, scheinen seine Arbeiten zu Lebzeiten auch von japanischer Seite her rege verfolgt worden zu sein, zumal Specht Ehrenmitglied des Verbands japanischer Psychiater war. Wenn die fachpsychiatrische Diskussion, ob die unipolare Manie eine spezielle Unterform der bipolar affektiven Störung oder womöglich eine abgegrenzte nosologische Entität darstellt, weiterhin nicht zu einer eindeutigen Antwort gefunden hat (vgl. [81]), so könnte sich eine Reevaluation von Spechts strenger Trennung des sekundären manischen Dauerzustandes von der echten chronischen Manie (vgl. [26, 40, 54]) durchaus lohnen.

Im Jahr 1998 schrieb Schmidt-Degenhard der Paranoiaauffassung der Erlanger Schule einen „richtigen Kern“ [44] zu: „wenn wir das Wesen der manischen Auslenkung bzw. der depressiven Phase in elementaren und polaren Verschiebungen des Selbstgefühls erblicken, so wird ersichtlich, dass der Pathologie des Selbstwertgefühls in der Dynamik der Paranoia eine zentrale Bedeutung zukommt“ [44]. Die aktuell wenig diskutierte Paranoiafrage verweist auf ein Dilemma der allgemeinen Psychopathologie: „Letztlich geht es hier um die Frage nach Wahnsinn und Verrücktsein und ihrer Bedeutung für das Selbstverständnis der Psychiatrie“ [44]. Nach Kraepelin ist die Geschichte des Paranoiabegriffes aufs engste mit der gesamten Entwicklung der psychiatrisch-klinischen Anschauungen verknüpft (vgl. [49]). Schmidt-Degenhard begründete das mangelnde zeitgenössische fachpsychiatrische Paranoiainteresse mit einer weitgehenden Vernachlässigung des allgemein-psychopathologischen Diskurses. Dieser schien damals schon beinahe auf operational-metrische Diagnostik zur Optimierung der Klassifikationsforschung reduziert zu werden. Daher sah Schmidt-Degenhard in der Paranoiafrage „eher ein nosologisches Scheinproblem“ [44], die nur partiell eine nosologische Thematik beinhalte. Vielmehr spiegle die Paranoiafrage ein zentrales Problem der allgemeinen Psychopathologie und womöglich auch der empirischen und philosophischen Anthropologie wider: Das Wähnen des Wahnkranken führt zur radikalen Abwendung zwischenmenschlicher Reziprozität. Um dem paranoischen Menschen in seiner Not gerecht zu werden, sollte der phänomenologisch-anthropologisch geschulte Psychopathologe in interdisziplinärem Dialog mit den verwandten Sozial‑, Geistes- und Kulturwissenschaften stehen.

Als Wegweisend für unser heutiges Krankheitsverständnis der emotional instabilen Persönlichkeitsstörung vom Borderline-Typ (vgl. [77]) könnte – unter Beachtung oben genannter Limitationen bei retrospektiver Diagnostik – Spechts Werk „Ueber Hystermelancholie“ [56] gelten. Specht sah zu viel Theoretisches in der Hysteriefrage kultiviert, wobei anzumerken ist, dass sich diese funktionelle Neurose in den damaligen medizinischen Kategorien zusammensetzte aus einer Konstellation folgender Symptome: Stimmungs- und Charakteranomalie, Krampfanfälle, sensorische und sensible Störungen, schmerzhafte Druckpunkte sowie Lähmungen mit oder ohne Kontrakturen (vgl. [45]).

Wird heutzutage der Selbstverletzungstendenz bei Patienten mit emotional instabiler Persönlichkeitsstörung vom Borderline-Typ u. a. durch sog. Skills zur Verminderung des Schneidedrucks begegnet, so vertrat Specht bei den hysteromelancholischen Patienten die therapeutische Devise „allein außergewöhnliche Verhältnisse erfordern außergewöhnliche Massnahmen“ [56]. So trat nach mechanischer Beschränkung „prompte psychische Beruhigung“ [56] bei Spechts Patienten ein.

Specht zeigt sich also generell Zwangsmaßnahmen gegenüber nicht abgeneigt. So hält er auch die Isolierung „[m]it Auswahl, in richtiger Weise angewandt […] oft für einen Segen für alle Teile“ [71]. Auch über das Dauerbad solle nach Specht „nicht der Stab gebrochen sein; auch wir in Erlangen machen von ihm viel Gebrauch, trotzdem es jetzt […] [1930] wieder einmal in Ungnade gefallen ist“ [71]. Ebenso könne man die Zellisolierungen „so in Bausch und Bogen“ [71] „nicht zum alten Eisen werfen“ [71].

Heutzutage könnte eine Fixierung bei Schneidedruck allenfalls dann näher erwogen werden, wenn der Borderline-Patient dies ausdrücklich wünscht, wobei sorgfältige Aufklärung und Dokumentation unerlässlich sind (vgl. [18]).

Ein in vielen Fällen hervorstechendes Symptom sei „die Selbstmordneigung in der Form des anhaltend betätigten Selbstvernichtungstriebes. Wo dieses Symptom sich zeigt, da darf man zu allererst an Hysteromelancholie denken. Haben wir bei anderen Melancholieformen immer mit dem gelegentlichen oder verstohlenen Auftreten dieser Gefahr zu rechnen, so tritt sie uns hier in fortgesetzten Alarmierungen aufdringlich entgegen und gestaltet sich umso unangenehmer, als man ihr gegenüber sich geradezu ratlos fühlen kann“ [56]. Ganz besonders hob Specht hervor, „dass diese Kranken tatsächlich schwer leiden“ [56]. Specht betonte, der Schmerz der Patienten sitze tief und er wollte das Krankheitsbild auf der Basis breiter klinischer Beobachtung geschildert wissen. Spechts Vertrauen darauf, dass die „Krankheitsform der Hysteromelancholie […] die ihr gebührende klinische Stellung definitiv erhalten“ [56] wird, könnte – unter der Einschränkung retrospektiver Diagnostik – wohl am ehesten seine Erfüllung gefunden haben in der aktuellen diagnostischen Einheit der emotional instabilen Persönlichkeitsstörung vom Borderline-Typus.

## Schlussfolgerung

Fundiertes psychopathologisches Wissen und interdisziplinärer Austausch tragen dazu bei, der psychischen Störung des Patienten als Individuum möglichst gerecht zu werden. Das Schlusswort gebührt Specht selbst mit einer Aussage, die uns die Wichtigkeit eines adäquaten interdisziplinären psychopathologischen Diskurses verdeutlicht, denn

„Was sich uns im hellen Licht des unmittelbaren Erlebens bietet, sind psychopathische Bilder; sie sind unser spezifisches Forschungsgebiet. Ihre Unterscheidung und Zusammenfassung bleibt unsere allererste Aufgabe auch dann, wenn wir einmal dem idealen Abschluss unserer Disziplin näher gerückt sein sollten“ [70].
